# The global prevalence of osteoporosis in the world: a comprehensive systematic review and meta-analysis

**DOI:** 10.1186/s13018-021-02772-0

**Published:** 2021-10-17

**Authors:** Nader Salari, Hooman Ghasemi, Loghman Mohammadi, Mohammad hasan Behzadi, Elham Rabieenia, Shamarina Shohaimi, Masoud Mohammadi

**Affiliations:** 1 Department of Biostatistics, School of Health, Kermanshah University of Medical Sciences, Kermanshah, Iran; 2 Student Research Committee, Kermanshah University of Medical Sciences, Kermanshah, Iran; 3 Department of Statistics, Science and Research Branch, Islamic Azad University, Tehran, Iran; 4 Department of Biology, Faculty of Science, University Putra Malaysia, Serdang, Selangor Malaysia; 5 Department of Nursing, School of Nursing and Midwifery, Kermanshah University of Medical Sciences, Kermanshah, Iran

**Keywords:** Prevalence, Osteoporosis, Meta-analysis, Systematic review

## Abstract

**Background:**

Osteoporosis affects all sections of society, including families with people affected by osteoporosis, government agencies and medical institutes in various fields. For example, it involves the patient and his/her family members, and government agencies in terms of the cost of treatment and medical care. Providing a comprehensive picture of the prevalence of osteoporosis globally is important for health policymakers to make appropriate decisions. Therefore, this study was conducted to investigate the prevalence of osteoporosis worldwide.

**Methods:**

A systematic review and meta-analysis were conducted in accordance with the PRISMA criteria. The PubMed, Science Direct, Web of Science, Scopus, Magiran, and Google Scholar databases were searched with no lower time limit up till 26 August 2020. The heterogeneity of the studies was measured using the *I*^2^ test, and the publication bias was assessed by the Begg and Mazumdar’s test at the significance level of 0.1.

**Results:**

After following the systematic review processes, 86 studies were selected for meta-analysis. The sample size of the study was 103,334,579 people in the age range of 15–105 years. Using meta-analysis, the prevalence of osteoporosis in the world was reported to be 18.3 (95% CI 16.2–20.7). Based on 70 studies and sample size of 800,457 women, and heterogenicity *I*^2^: 99.8, the prevalence of osteoporosis in women of the world was reported to be 23.1 (95% CI 19.8–26.9), while the prevalence of osteoporosis among men of the world was found to be 11.7 (95% CI 9.6–14.1 which was based on 40 studies and sample size of 453,964 men.). The highest prevalence of osteoporosis was reported in Africa with 39.5% (95% CI 22.3–59.7) and a sample size of 2989 people with the age range 18–95 years.

**Conclusion:**

According to the medical, economic, and social burden of osteoporosis, providing a robust and comprehensive estimate of the prevalence of osteoporosis in the world can facilitate decisions in health system planning and policymaking, including an overview of the current and outlook for the future; provide the necessary facilities for the treatment of people with osteoporosis; reduce the severe risks that lead to death by preventing fractures; and, finally, monitor the overall state of osteoporosis in the world. This study is the first to report a structured review and meta-analysis of the prevalence of osteoporosis worldwide.

## Background

Osteoporosis is a common disease all over the world. Osteoporosis has been operationally defined based on bone mineral density (BMD) assessment. According to the WHO criteria, osteoporosis is defined as a BMD that lies 2.5 standard deviations or more below the average value for young, healthy women (a *T*-score of <  − 2.5 SD) (1, 6). The most widely validated technique to measure BMD is dual-energy X-ray absorptiometry (DXA), and diagnostic criteria based on the *T*-score for BMD area recommended entry criterion for developing pharmaceutical interventions in osteoporosis (7–9) [1].

Osteoporosis is classified as primary (includes type I and type II) and secondary. Primary osteoporosis is seen in post-menopausal women and men and women over 70 years of age due to ageing [2]. Secondary osteoporosis is caused by diseases, treatments or idiopathic. Systemic diseases, endocrine diseases, and malignant neoplasms are among the diseases that cause secondary osteoporosis. Besides, chronic use of glucocorticoids, lifestyle conditions, habits, and major depression are other causes of osteoporosis [2].

Various methods are used to measure osteoporosis. Typically, to diagnose osteoporosis, bone mineral density (BMD) is measured by dual-energy X-ray absorptiometry (DXA) at various skeletal sites [3]. Another way to diagnose osteoporosis is the speed of sound (SOS) in the tibia, which can be measured by ultrasound imaging [4].

Risk factors for osteoporosis are divided into two categories: modifiable and non-modifiable [5]. Weight, smoking, alcohol consumption [6], physical inactivity, dietary calcium deficiency, and long-term glucocorticoid use are among the risk factors for the modifiable osteoporosis group. Gender, age, race, and genetic characteristics are among the risk factors for the non-modifiable osteoporosis group [5]. These factors can also be more widespread with respect to gender. For example, in women, premature menopause and loss of ovarian function before menopause are other risk factors for osteoporosis [6].

A study in Turkey showed that women between the ages of 18–49 who smoke, have fair skin, or have a family history of osteoporosis are at higher risk for osteoporosis [7]. The clinical symptoms of osteoporosis in old age include decreased body height, dowager’s hump or kyphosis, bone fracture and respiratory impairment [8].

In a double-blind placebo-controlled study in osteoporosis comprised of 483 women with post-menopausal osteoporosis, 110 women with secondary osteoporosis, and 84 men with osteoporosis of any cause, aged between 28 and 88 years old, the mortality rate in people with one or more fractures was 4.4 times higher [9]. The incidence of osteoporotic fractures has made it one of the leading causes of death in the elderly [3]. Because the risk of osteoporotic fractures is higher in older women than in older men, all menopausal women should be screened for signs of osteoporosis [10]. Fractures usually occur in three areas: vertebrae, distal arm, and hip [11].

Vertebral fractures are more common in women than men [11]. Research has shown that if women have to be divided into three groups; premenopausal (before menopause), the onset of menopause, and women with over five years of menopause, bone fractures due to osteoporosis were more common in post-menopausal women than in premenopausal women or around the onset of menopause [12]. Twenty per cent of women die within a year of a fracture [11].

Men have more bone mass during growth and develop more muscle mass, which provides more skeletal integration. Men do not experience menopause. Also, they have a shorter life expectancy than women; therefore, less time is available to develop the disease. The prevalence of osteoporosis in older men than in young men is also based on this fact [11].

Osteoporosis is a problem for both sexes. However, the majority of research on osteoporosis has focused on women because women are more likely than men to develop osteoporosis and subsequent fractures [11]. So far, many studies have been conducted on the prevalence of osteoporosis in different parts of the world. These studies have either been based on small samples from the target population [13], or to a lesser extent, based on all data collected in the medical databases of a country such as the USA [14] and Korea [15]. According to a study based on the SOS criteria in 2003, the prevalence of osteoporosis in Chinese women was reported to be 10.08% [4]. In another study in 2005, the prevalence of osteoporosis in Vietnamese women, based on the BMD criteria, was reported to be 15.4% [16].

Orthopaedic surgeons are typically only involved in the osteoporotic patient’s care as a consequence of a fracture and with the single biggest risk factor for a future fracture being a previous fragility fracture, it, therefore, follows that the area of focus for the orthopaedist should be on the secondary prevention of future fractures [9, 10]. With the instigation of the Own the Bone program by the AOA, the idea of the orthopaedist being a key component in the care of a patient’s bone health, beyond the acute fracture care, has gained a great deal of traction [10, 11].

Interestingly enough, one of the fractures that is the most common in the osteoporotic individual is also the most often missed: vertebral body fractures. They are most often missed due to a lack of inclusion in the differential diagnosis of patients with back pain and are thus overlooked [11–14]. A vertebral body fracture should be suspected in any patient at risk for osteoporosis with back pain or kyphosis [11–14].

Studies in many different countries have demonstrated that with increased communication between the orthopaedist, patient and patient’s PCP, there is increased usage of pharmacotherapeutics, calcium and vitamin D supplementation, and BMD assessment with DXA scan [14–17]. There is also good evidence that the use of calcium, vitamin D and pharmaceutical interventions results in a decreased risk of fragility fractures [14–17].

A study of 773 Indian men and women between the ages of 30 and 90 showed that the prevalence of osteoporosis was 24.7%. The prevalence in women was reported to be 15%; 10.3% was related to post-menopausal women, and 4.7% to premenopausal women. In this study, the prevalence in men was reported to be 9.7% [17].

The prevalence of osteoporosis in a sample of 524 Indian people between the ages of 20 and 85 was reported to be 6.9%, 11.1% of which were women, and 3.9% were men [18].

According to the data taken in a random sample from the Taiwan National Health Insurance (NHI) database in 2006, the prevalence of osteoporosis in Taiwanese men over the age of 50, based on BMD criteria, was reported to be 1.63% [19]. While in a survey in 2018, this rate was reported at 9.7% [19–21].

In another study, the prevalence of osteoporosis in Saudi Arabia men between the ages of 30 and 90 years was reported to be 24.1%; 19.2% of which was related to the age range of 30–50 years and 23.5% was related to the age range of 50–90 years [21].

These discrepancies in reports of the prevalence of osteoporosis can be seen in research in other parts of the world.

It is important to have consistent information on the prevalence of osteoporosis worldwide. With increasing life expectancy and longevity, the prevalence of osteoporosis and related fractures is increasing [15]. This is a serious challenge not only for health officials but also for individuals and their families and society in general [15]. Determining the prevalence and incidence of osteoporotic fractures is the first step in adopting the necessary strategies to reduce the burden of this challenge and concerns [15]. Due to the dispersion of reports related to the prevalence of osteoporosis in the world, which was based on small and large samples, and also lack of estimates of the prevalence worldwide, we decided to have a systematic review of all studies conducted in this field and examined the worldwide prevalence of osteoporosis, using meta-analysis tools.

Therefore, this study aims to investigate the systematic analysis of evidence and studies to report the prevalence of osteoporosis worldwide.

## Methods

### Search strategy and study selection procedure

Searches in this meta-analysis study were performed by two researchers. As part of the research methodology, PubMed, Science Direct, Web of Science, Scopus and Persian language databases such as SID and Magiran were searched with limited English and Persian language and no time limit until August 2020. The keywords used to search for resources were selected from the Medical Subject Headings (MeSH) database in this study. A search using keywords osteoporosis, osteoporosis, prevalence, cross-sectional, age-related, post-traumatic, and all the possible combinations of these words were designed according to the pattern of each database. All information related articles were identified and added to the EndNote bibliography management software. In addition to maximize the comprehensiveness of the search, the lists of references in the identified articles were manually reviewed. After collecting articles, the duplicate papers that were identified within various databases were excluded.

Search strategy in all databases: ((((((((osteoporosis [Title/Abstract]) OR Age-Related Osteoporosis [Title/Abstract]) OR Bone Loss [Title/Abstract]) OR Post-Traumatic [Title/Abstract]) OR Senile Osteoporosis [Title/Abstract]) AND prevalence OR Period Prevalence OR Point Prevalence)))))))

Inclusion criteria were as follows: Studies that have examined the prevalence of osteoporosis, observational (cross-sectional) studies, and studies whose full text was available.

Exclusion criteria were as follows: Duplicate studies, unrelated studies to the subject and purpose of this study, unclear methodology, interventional studies, case report studies, studies whose full text was not available, and studies whose language was not Persian or English.

### Study selection procedure

Initially, all articles related to osteoporosis were collected, and a list of abstracts was prepared after the search was completed. At this point, all articles titled ‘Prevalence’ and ‘Osteoporosis’ entered the initial list. Then, a checklist appropriate to the type of study was used, which includes author’s name, title, year and month of publication, place of study, sample size, the overall prevalence, and risk factors for all studies that were initially evaluated were prepared for final evaluation. Accordingly, the full text of the remaining articles from the previous stage, i.e. screening, were carefully examined, and irrelevant studies were excluded by considering the inclusion and exclusion criteria. In order to prevent bias, all stages of resource review and data extraction were performed by two reviewers independently. If an article was not included, the reason for the exclusion was mentioned. Then, those articles that included patients with osteoporosis were finally approved. In the end, 86 relevant articles entered the meta-analysis stage. The full text of the articles was reviewed for final analysis.

### Quality evaluation

To evaluate the quality of articles (i.e. methodological validity and results), a checklist appropriate to the type of study was used. STROBE checklists are commonly used to critique and evaluate the quality of observational studies, such as the present study. The STROBE checklist consists of six scales/general sections: title, abstract, introduction, methods, results, and discussion. Some of these scales have subscales, resulting in a total of 32 subscales/items. Accordingly, the maximum score that could be obtained using the STROBE 32 checklist is 32 [20]. Considering the score of 16 as the cut-off point, articles with scores of 16 or above were considered medium- or high-quality articles. Furthermore, articles with scores below 16 were considered weak- or low-quality articles and excluded from the study. In the present study, based on the evaluation conducted using the STROBE checklist, 86 articles were entered for the systematic review and meta-analysis process.

### Statistical analysis

Since the prevalence rate has a binomial distribution, the variance of the prevalence was calculated using the binomial distribution formula, and the average weight was used to combine the prevalence rate in different studies. As well as to evaluate the heterogeneity of the selected studies, the *I*^2^ index was used. Therefore, the random effects model was used to combine the results of the studies. Meta-regression was used in order to investigate the relationship between the prevalence of osteoporosis and the year of study and sample size. To investigate the publication bias, due to the high volume of samples entered into the study, the Begg and Mazumdar’s test and corresponding Funnel plots were adopted with a significance level of 0.1. Data analysis was performed using the Comprehensive Meta-Analysis (Version 2) software.

## Results

**Fig. 1:**
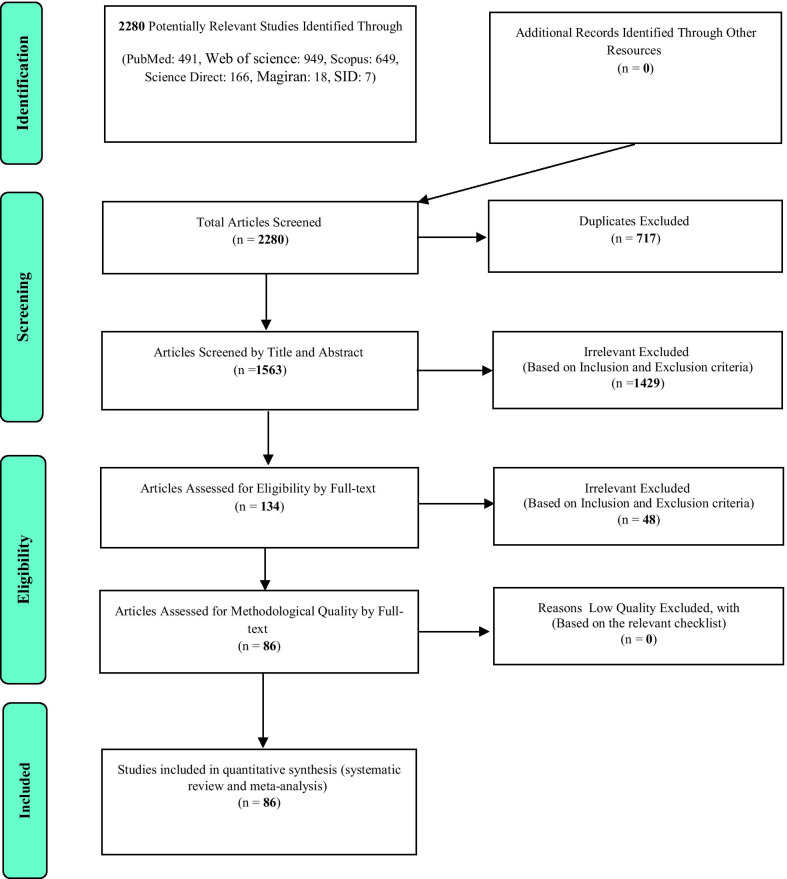
The flow chart on the stages of including the studies in the systematic review and meta-analysis (PRISMA 2009)

As shown in Fig. 1 and based on the initial search in the database, 2280 articles were found, of which 491 articles were extracted from the PubMed database, 166 articles from the Science Direct database, 949 articles from Web of Science, 649 articles from Scopus, seven articles from SID, and 18 articles from Magiran. Out of the total number of articles, 717 articles were duplicates that were excluded in the first stage. In the screening stage, 1429 articles were excluded by considering the inclusion and exclusion criteria and the application of time limit from 2000 to 2020. Eight articles that seemed to be related to the study were excluded from the study due to the lack of access to their full text. In the eligibility evaluation stage, the full texts of the remaining 134 articles were examined based on the inclusion and exclusion criteria, and 48 irrelevant articles were omitted. The studies were reviewed based on the four-step process of PRISMA2009 (Fig. 1), including identifying articles, screening, reviewing the criteria for accepting articles, and the articles that entered the meta-analysis process. Finally, 86 articles were included in the final analysis; their information is given in Table 1.

**Table 1:** Summary of characteristics of included studies

Row number	Author	Year	Continent	Country	Diagnosis tool	Location of diagnosis	Cut-off	Study population	*m*	*w*	Age	OP
1	Hyun Koo Uoon [22]	2001	Asia	Tae-An Korea	BUA by QUS2	Calcaneus	*T* ≤ − 2.5	298	0	298	35–65	9
2	Sireen Shilbayeh [23]	2003	Asia	Jordan	BMD by DXA	Femoral neck, lumbar spine	*T* ≤ − 2.5	400	0	400	19–89	119
3	X.-P. WU [4]	2003	Asia	China	SOS	Tibial	*T* ≤ − 2.5	1596	0	1596	46.5	173
4	Vu Thi Thu Hien [16]	2005	Asia	Vietnam	SOS	Calcaneus	*T* ≤ − 3.8	2232	0	2232	≥ 20	343
5	Sarath Lekamwasam [24]	2006	Asia	Sri Lanka	BMD by DXA	Spine, femoral neck	*T* ≤ − 2	1642	0	1642	≥ 50	736
6	G. Chhibber [25]	2006	Asia	Dehli and Haryana (INDIA)	BMD by DXA	Forearm, hip	*T* ≤ − 2.5	430	0	430	60–80	265
7	Mahmoud I. El-Desouki [21]	2007	Asia	Saudi Arabia	BMD by DXA	Lumbar spine, femoral neck	*T* ≤ − 2.5	429	429	0	30–90	101
8	Nan-Ping Yang [19]	2006	Asia	Taiwan	BMD by DXA	Lumbar, hip	*T* ≤ − 2.5	33,633	17,583	16,050	≥ 50	2109
9	Abdulbari Bener [26]	2007	Asia	Qatar	BMD by DXA	Spine, femur	*T* ≤ − 2.5	821	0	821	20–70	42
10	Didem Arslantas [27]	2008	Asia	Turkey	BMD by DXA	Hip	*T* ≤ − 1.8	1437	571	866	40–89	216
11	Montchai Chumnumnawin [28]	2008	Asia	Bangkok-Thaiwan (Priests)	BMD by DXA	Hip	*T* ≤ − 2.5	659	659	0	≥ 20	33
12	S. Miura [29]	2008	Asia	Philippines	SOS	Calcaneus	*T* ≤ − 1.79	339	0	339	40–89	67
13	P. Shokrollahi [30]	2008	Asia	IRAN, SHIRAZ	BMD by DXA	BMD		75	0	75	≥ 55	58
14	M. Fatima [31]	2009	Asia	Pakistan	BMD by SOS	Calcaneus	*T* ≤ − 2.5	334	0	334	20–60	43
15	Sarath Lekamwasam [32]	2009	Asia	Sri Lanka	BMD by DXA	Middle phalanx of the middle finger of the non-dominant hand	*T* ≤ − 2.5	1147	1147	0	50–84	66
16	Aranjan Lionel Karunanayake [33]	2010	Asia	Sri Lanka	BMD by DXA	Lumbar spine, hip	*T* ≤ − 2.5	700	279	421	35–64	101
17	A. Neema [34]	2010	Asia	Wardha, India	BMD by SOS	Calcaneus	*T* ≤ − 1.8	1122	0	1122	40–60	173
18	Shafaq Zahoor [35]	2010	Asia	Pakistan	BMD by DXA	Heel	*T* ≤ − 2.5	240	0	240	≥ 49	56
19	Neelam Aggarwal [36]	2011	Asia	Chandigarh, India	BMD by DXA	Lumbar spine, femoral neck, and total spine	*T* ≤ − 2.5	200	0	200	≥ 45	56
20	Zhifeng Sheng [37]	2011	Asia	Chine	BMD by DXA	Lumbarspine, left femoral	*T* ≤ − 2.5	954	0	954	50–82	376
21	Yong Jun Choi [38]	2012	Asia	Korea	BMD by DXA	Lumbar spine, total femur, femur neck	*T* ≤ − 2.5	4946	2095	2851	≥ 50	1169
22	Kyae Hyung Kim [39]	2012	Asia	Korea	BMD by DXA	Lumbar spine, femoral neck	*T* ≤ − 2.5	2870	0	2870	≥ 50	1122
23	Zhang Mengmeng [40]	2012	Asia	Changchun, China	BMD by DXA	Distal, forearm	*T*	16,019	7286	8733	20–89	4313
24	Zahra Pourhashem [41]	2012	Asia	Amirkola, Iran	BMD by DXA	Femur, spine, femoral, lumbar	*T* ≤ − 2.5	193	105	88	60–88	62
25	S. Tuzun [42]	2012	Asia	Turkey	BMD by DXA	Lumbar spine, proximal femur (neck, total), femoral neck	*T* ≤ − 2.5	1965	944	1021	≥ 50	202
26	Neeraj Kumar Agrawal [43]	2013	Asia	India	BMD by DXA	Right femur, neck, trochanter, total hip	*T* ≤ − 2.5	200	200	0	≥ 50	17
27	Maj Tripti Agrawal [44]	2013	Asia	India	BMD by QUS	Calcaneus (heel)	*T* ≤ − 2.5	158	0	158	35–64	21
28	Maninder Kaur [45]	2013	Asia	North, India	BMD by DXA	Lumbar spine	*T* ≤ − 2.5	250	0	250	45–80	66
29	Jongseok Lee [46]	2013	Asia	Korea	BMD by DXA	Femoral neck, lumbar spine	*T* ≤ − 2.5	17,205	7837	9368	10_89	4077
30	Yaşar Keskin [47]	2014	Asia	Turkey	BMD by MetriScan device	Middle phalanges of the second, third, and fourth digits of the non-dominant hand	*T* ≤ − 2.5	620	122	498	40–89	88
31	Kyung-Shik Lee [48]	2014	Asia	Korea	BMD by DXA	Total hip, femoral neck, total lumbar spine	*T* ≤ − 2.5	11,142	5355	5787	≥ 50	2557
32	Eun Jung Park [49]	2014		Korea	BMD by DXA	Lumbar spine, femoral neck, trochanter, total hip	*T* ≤ − 2.5	7425	3414	4011	≥ 50	1773
33	Edith Ming Chu Lau [50]	2015	Asia	China	BMD by DXA	Lumbar spine, total hip, femoral neck	*T* ≤ − 2.5	12,401	0	12,401	50–89	2798
34	Cathy Nga Yan Lee [13]	2015	Asia	Hong Kong	BMD by DXA	Heel	*T* ≤ − 2.5	80	22	58	41.6	3
35	Zahra Mohammadi [51]	2015	Asia	Kurdistan, Iran	BMD by DXA	Lumbar spine, hip, femoral neck	*T* ≤ − 2.5	306	403	629	≥ 50	123
36	Marzieh Saei Ghare Naz [52]	2015	Asia	Urmia, Iran	BMD by DXA	Femoral neck, lumbar spine	*T* ≤ − 2.5	292	0	292	≥ 50	152
37	Yan-Jiao Wang [53]	2015	Asia	China	BMD by DXA	Lumbar spine, femoral neck	*T* ≤ − 2.5	316	164	152	≥ 65	78
38	Khurshid A. Bhat [54]	2018	Asia	INDIA	BMD by DXA	Lumbar, total hip, femur neck	*T* ≤ − 2.5	241	241	0	≥ 60	46
39	Yi-Chien Lu [55]	2016	Asia	Taiwan, China	BMD by DXA	Lumbar spine, femoral neck, both	*T*_usa ≤ − 2.5	3740	2028	1712	≥ 50	886
							*T*_Asia ≤ − 2.5					271
40	Sung Bae Park [15]	2016	Asia	Korea	BMD by DXA	Spine, hip, or wrist	*T* ≤ − 2.5	51,169,141	–	–	All	2,018,236
41	Sung Bae Park [15]	2016	Asia	Korea	BMD by DXA	Spine, hip, or wrist	*T* ≤ − 2.5	50,908,646	–	–	All	2,018,437
42	Dana Hyassat [56]	2017	Asia	Amman, Jordan	BMD by DXA	Total, lumbar spine, left femoral neck	*T* ≤ − 2.5	1079	0	1079	45–84	405
43	Yu-Jun Kwon [57]	2017	Asia	Korea	BMD	Heel	*T* ≤ − 2.5	595	157	438	51–94	393
44	Gul Pinar [7]	2017	Asia	Turkey	BMD by DXA	Femoral neck, lumbar spine	*T* ≤ − 2.5	1792	0	1792	18–49	72
45	Limin Tian [58]	2017	Asia	Northwestern of China	BMD by DXA	Distal one-third radius of the forearm	*T* ≤ − 2.5	6564	3205	3359	≥ 40	583
46	Muhammad Farhan Abbas [59]	2018	Asia	Pakistan	BMD by X-rays	Questionnaire	*T* ≤ − 2.5	360	0	360	≥ 15	152
47	Parvin Cheraghi [60]	2018	Asia	Hamedan, Iran	BMD by DXA		*T* ≤ − 2.5	1779	1077	702	≥ 60	142
48	Nidhi S. Kadam [61]	2018	Asia	Pune City, India	BMD by DXA	Lumbar spine, femoral neck, total hip	*T* ≤ − 2.5	421	193	228	40–75	69
49	Neelam Kaushal [18]	2018	Asia	INDIA	BMD by DXA	Lumbar spine, femur neck, total femur	*T* ≤ − 2.5	524	306	216	20–85	36
50	Chi‐Hua Ko [20]	2018	Asia	Taiwan	BMD by DXA	Hip (total), lumbar spine, femoral neck	*T* ≤ − 2.5	3734	3734	0	≥ 50	362
51	P. Modagan [17]	2018	Asia	INDIA	BMD by DXA	Proximal femur (total hip, femoral neck, shaft, Ward’s triangle, trochanter), anteroposterior (AP) lumbar spine	*T* ≤ − 2.5	773	380	393	30–90	191
52	Nayer Seyfizadeh [62]	2016	Asia	Iran	BMD by DXA	Lumbar spine, femoral neck	*T* ≤ − 2.5	990			55–92	307
53	Jung Eun Yoo [63]	2018	Asia	Korea	BMD by DXA	Femoral neck, total femur, lumbar spine	*T* ≤ − 2.5	6104	6104	0	≥ 30	305
54	Abdulaziz Ahmed Abdulaziz [64]	2019	Asia	SAUDI ARABIA	BMD by DXA	Lumbar spine, neck femur	*T* ≤ − 2.5	131	131		≥ 60	34
55	Zaheer Ahmed Mohammed [64]	2019	Asia	MAJMAAH, Saudi	BMD by DXA	Hip, spine (online questionnaire)	*T* ≤ − 2.5	593	110	483	≥ 20	47
56	K. Padmanabhan [65]	2019	Asia	Chennai, India	BMD by DXA	Calcaneus heel	*T* ≤ − 2.5	270	0	270	30–70	43
57	Hasanga Rathnayake [66]	2019	Asia	Sri Lanka	BMD by DXA	Spine, femoral neck, total hip	*T* ≤ − 2.5	176	0	355	≥ 50	65
58	Shriraj Shrestha [67]	2019	Asia	Hospital in Nepal	BUA and SOS by QUS	Centre of the bone	*T* ≤ − 2.5	464	141	323	41.02	38
59	Shaanthana Subramaniam [68]	2019	Asia	Malaysia	BMD by DXA	Lumbar spine, total hip	*T* ≤ − 2.5	367	182	185	≥ 40	56
60	Peizhi Wang [69]	2019	Asia	Singapore	Self-assessment tool	Self-assessment tool	High-Risk Index (female) < − 4, High-Risk Index (male) < − 6	2345	1052	1293	60–105	1218
61	Qiang Zeng [70]	2019	Asia	China	BMD by DXA	Lumbar spine, Femoral neck, Total femur	*T* ≤ − 2.5	41,347	40,944	34,377	≥ 50	7211
62	Kyeong Jin Kim [71]	2020	Asia	Korea	BMD by DXA	Lumbar spine, femur neck, total hip	*T* ≤ − 2.5	208	0	488	≥ 50	52
63	Mamatov Sagynali Murzaevich [72]	2020	Asia	Kyrgyz	BMD by ultrasound bone densitometer		*T* ≤ − 2.5	1200	509	691	18–79	179
64	Qian Zhang [8]	2020	Asia	Shanghai, China	BMD by DXA	Proximal femur, lumbar vertebrae	*T* ≤ − 2.5	565	231	334	70–95	223
65	Florent Richy [73]	2004	Europe	Belgium	BMD by DXA	Total femur, femoral neck, lumbar spine	*T* ≤ − 2.5	311	311	0	30–91	63
66	Eric Lespessailles [74]	2009	Europe	France	BMD by DXA	Hip, spine, wrist by face-to-face interviews	*T* ≤ − 2.5	2613	0	2613	≥ 45	254
67	Henrik G Ahlborg [75]	2010	Europe	Malmö, Sweden	BMD by single-photon	Distal radius, forearm	*T* ≤ − 2.5	459	0	459	≥ 50	69
68	Patrizia D’Amelio [76]	2013	Europe	Italy	BMD by DXA	Lumbar spine, femoral neck	*T* ≤ − 2.5	995	0	995	45–92	335
69	E. J. Marjanovic [77]	2013	Europe	UK	BMD by DXA	Proximal femur, lumbar spine	*T* ≤ − 2.5	380	0	380	45–65	98
70	Marıa-Jesus Gómez-de-Tejada Romero [78]	2013	Europe	Spain	BMD by DXA	Lumbar spine, femoral neck	*T* ≤ − 2.5	1229	0	1229	≥ 50	383
71	Loredana Cavalli [79]	2016	Europe	Italy	BMD by QUS	Heel	*T* ≤ − 2.5	7305	1191	6114	17–97	1212
72	Marie-Therese Puth [80]	2018	Europe	Germany	telephone survey	Self-reported		10,660	4961	5699	≥ 50	911
73	B. R. Nielsen [81]	2020	Europe	Denmark	BMD by DXA	Spine and hip	*T* ≤ − 2.5	529	232	297	≥ 65	101
74	Alexandre Faisal-Cury [6]	2007	America	Sao Paulo	BMD	Femur, hip	*T* ≤ − 2.5	999	0	999	50–96	320
75	Julie Robitaille [82]	2008	America	U.S.	By a physician in the household	Household interview	By a physician	8073	0	8073	≥ 20	442
76	H. Cheng [14]	2009	America	AMERICA	BMD by DXA	Medicare data		911,327	359,733	551,594	≥ 65	270,907
77	Arthur Swislocki [83]	2010	America	Nursing Home, United States	BMD by DXA	Lumbar spine, total hip, femoral neck	*T* ≤ − 2.5	106	106	0	≥ 50	33
78	John Londono [84]	2013	America	Colombia	BMD by DXA	Lumbar vertebrae, femur neck	*T* ≤ − 2.5	795	0	795	35–53	38
79	Robert Ferrari [85]	2015	America	Canada	BMD by DXA	Hip, lumbar spine	*T* ≤ − 2.5	557	557	0	65–75	6
80	Carlos Mautalen [86]	2016	America	Buenos Aires, Argentina	BMD by DXA	Lumbar spine, femoral neck	*T* ≤ − 2.5	5448	0	5448	≥ 50	1021
81	Sabrina E. Noel [87]	2018	America	USA	BMD by DXA	Femoral neck, lumbar spine	*T* ≤ − 2.5	953	273	680	47–79	100
82	Ricardo M. Lima [88]	2019	America	Brezil	BMD by DXA	Lumbar spine, femoral neck	*T* ≤ − 2.5	234	0	234	68.3	37
83	T. O. Alonge [89]	2017	Africa	Nigeria	BMD by DXA	Right wrist	*T* ≤ − 2.5	2401	964	1437	≥ 60	1366
84	P. O. Ezeonu [90]	2017	Africa	South-East Nigeria	BMD	Right calcaneal bone	*T* ≤ − 2.5	327	0	327	18–44	119
85	Fred Chuma Sitati [91]	2020	Africa	Kenya, African	BMD by DXA	Lumbar spine, hip	*T* ≤ − 2.5	254	0	254	50–95	67
86	E. P. Boschitsch [92]	2017	Australia	Australia	BMD by DXA	Hip, the distal forearm, vertebrae	*T* ≤ − 2.5	99,399	0	99,399	≥ 40	13,444

**Fig. 2:**
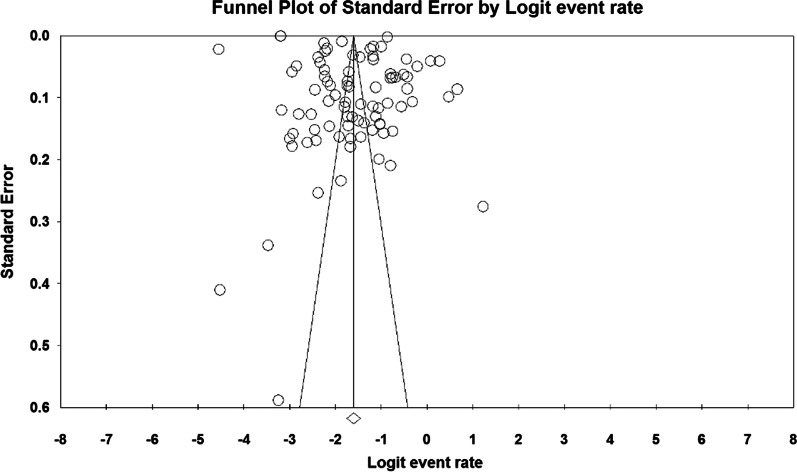
Funnel diagram of the result of the overall prevalence of osteoporosis worldwide

Due to the heterogeneity of the selected studies, the *I*^2^ test (*I*^2^ = 97.9) and the random effects model were used to combine the reported results of studies and approximate the total prevalence. However, according to the results of Begg and Mazumdar’s test and funnel diagram at a significance level of 0.1, no bias was observed in the results of the prevalence of osteoporosis worldwide in this study (*P* = 0.103) (Fig. 2).

### Meta-analysis

**Fig. 3:**
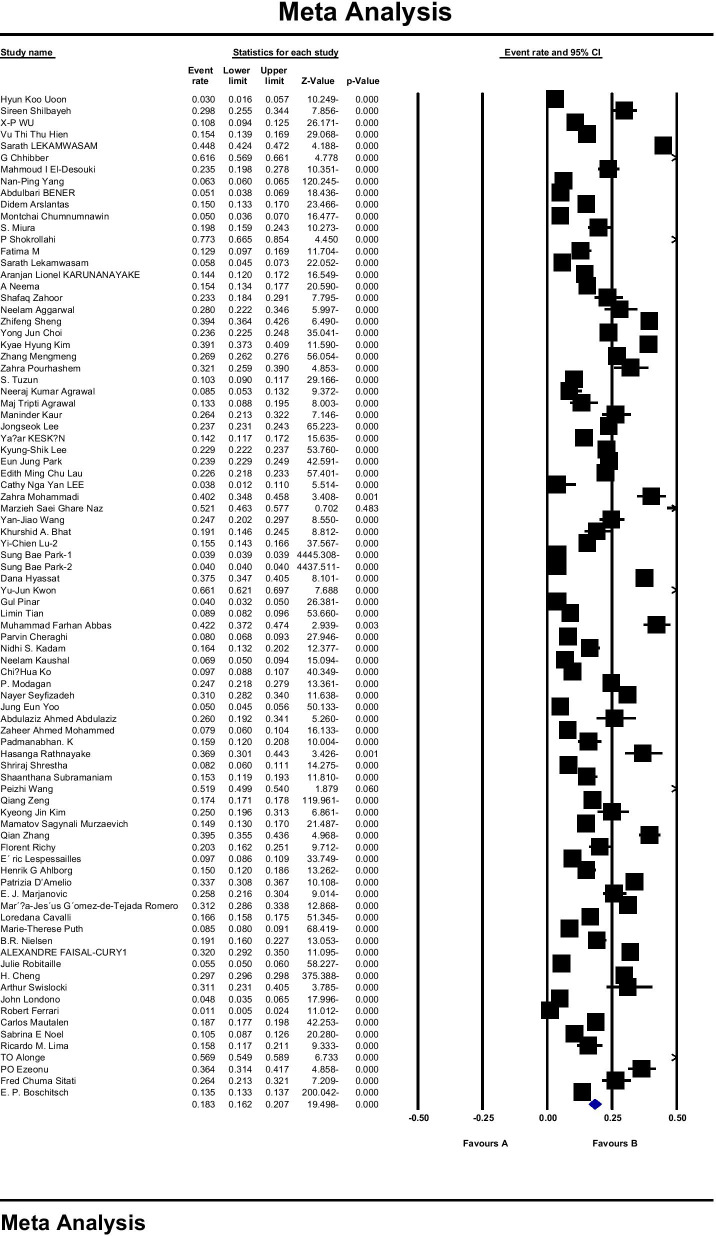
Overall prevalence of osteoporosis in the world based on a random effects model

A total of 86 studies were used to assess the prevalence of osteoporosis in the world, including 64 studies examining the prevalence of osteoporosis in Asian countries, nine studies in the European population, nine studies in the USA, three studies in Africa, and one study in Australia. The sample size was 103,334,579 people in the age range 15–105 years, and the prevalence of osteoporosis in the world was reported to be 18.3 (95% CI 16.2–20.7). The midpoint of each line segment indicates the prevalence in each study, and the diamond shape indicates the prevalence in the population for the entire study (Fig. 3).

In addition to reporting the prevalence of osteoporosis worldwide, the prevalence of this disease across five continents was also reported in this study. Table 2 shows the prevalence of osteoporosis in the world and by continent. Accordingly, the highest prevalence of osteoporosis was reported in Africa with 39.5% (95% CI 22.3–59.7). Based on the results of Begg and Mazumdar’s test at a significance level of 0.1, no bias was observed in the results prevalence of osteoporosis in the world and by continents (*P* > 0.05). However, the number of reported epidemiological studies on osteoporosis in Africa is limited. Based on the results of this study, it was revealed that the prevalence of osteoporosis in Africa is much worse than in other continents. The prevalence of osteoporosis in the Americas is far better than that in Europe and Asia. The prevalence of osteoporosis in Asia is higher than that in the USA and Australia. Likewise, the prevalence of osteoporosis in Asia is lower than in Africa and Europe.

**Table 2:** Results of meta-analysis by continents and diagnosis tools

Subgroup	Number of articles	Sample size	*I*^2^	Publication bias (Begg and Mazumdar test)	Prevalence % (95% CI)
*Continents*					
Asia	64	102,279,215	99.9	0.106	16.7 (95% CI 15.9–17.5)
Europe	9	24,481	99.1	1.000	18.6 (95% CI 12.9–26)
America	9	928,492	99.6	0.916	12.4 (95% CI 7.4–19.5)
Africa	3	2989	98.2	0.296	39.5 (95% CI 22.3–59.7)
Australia	1	99,399	100	–	13.5 (95% CI 13.3–13.7)
*Diagnosis tools*					
BMD (DXA)	71	102,398,640	99.9	0.112	19 (95% CI 18–20)
BMD by (DEXA)	11	923,401	99.3	0.533	19.6 (95% CI 14.3–26.2)
SOS	3	4116	92.2	1.000	14.8 (95% CI 10.9–19.7)

The results of prevalence of osteoporosis in terms of diagnostic tools are reported in Table 2, according to which the highest prevalence of osteoporosis with BMD instrument was 19.6 (95% CI 14.3–26.2).

### Prevalence of osteoporosis in women

**Fig. 4:**
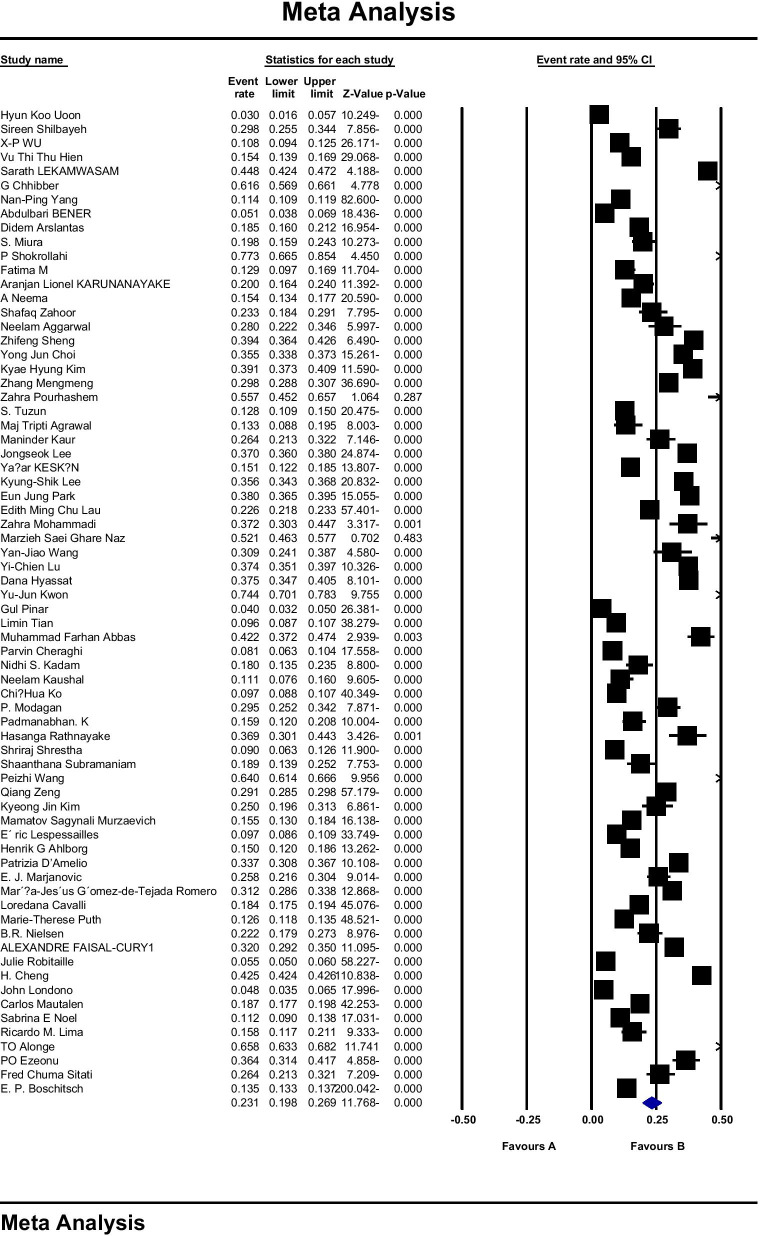
Overall prevalence of osteoporosis in women of the world based on a random effects model

In Fig. 4, based on 70 studies and sample size of 800,457 women and heterogenicity (*I*^2^: 99.8), the prevalence of osteoporosis in women of the world was reported to be 23.1 (95% CI: 19.8–26.9). According to the results of Begg and Mazumdar’s test at a significance level of 0.1, no bias was observed in the prevalence of osteoporosis in women of worldwide in this study (*P* = 0.227). The analysis of the results of the prevalence of osteoporosis by sex on each continent is reported in Table 3.

**Table 3:** Results of meta-analysis by continents stratified by sex

Continents (sex)	Number of articles	Sample size	*I*^2^	Begg and Mazumdar test	Prevalence % (95% CI)
*Asia*					
Men	31	85,636	99.3	0.414	11.7 (95% CI 8.8–15.5)
Women	51	113,431	99.3	0.188	24.3 (95% CI 21.2–27.8)
*Europe*					
Men	4	6695	98.1	0.308	9.7 (95% CI 4.4–18.5)
Women	8	17,786	98.7	0.710	19.8 (95% CI 14.5–26.5)
*America*					
Men	4	360,669	96.09	0.734	8.5 (95% CI 3.7–14.1)
Women	7	567,823	99.8	1.000	15.1 (95% CI 6.9–29.9)
*Africa*					
Men	–	–	–	–	–
Women	3	2018	98.9	0.296	42.4 (95% CI 19.9–56.5)

### Prevalence of osteoporosis in men

**Fig. 5:**
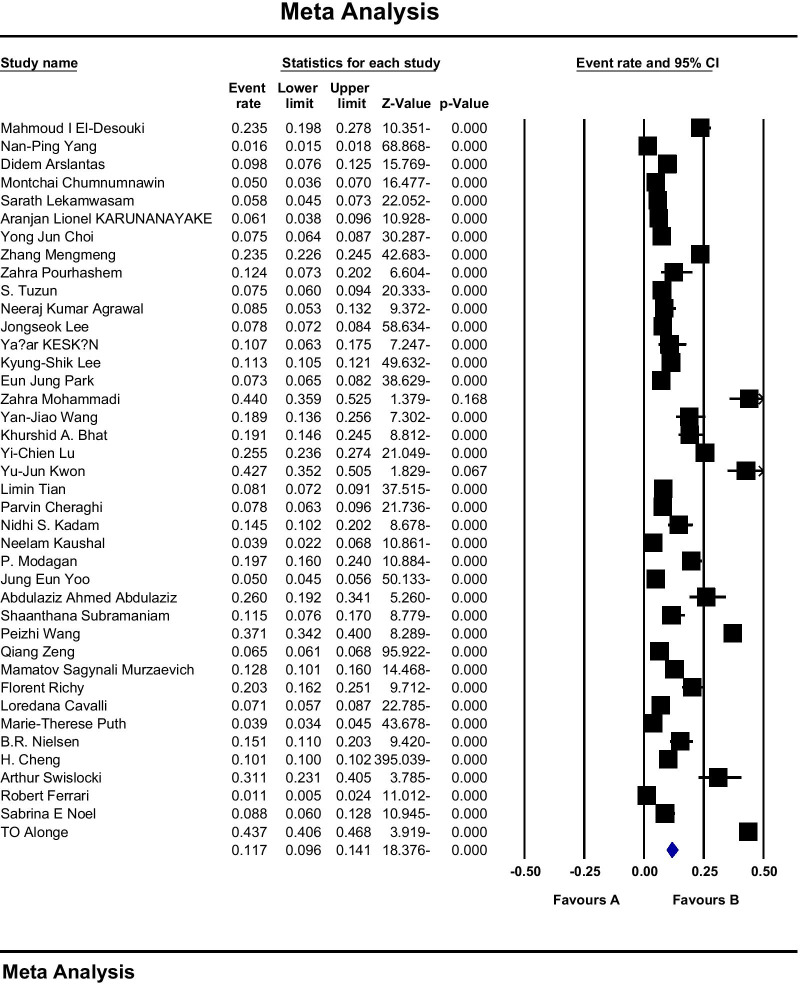
Overall prevalence of osteoporosis in men of the world based on a random effects model

In Fig. 5, based on 40 studies and sample size were 453,964 men and heterogenicity (*I*^2^: 99.3), the prevalence of osteoporosis in men of the world was reported to be 11.7 (95% CI 9.6–14.1). According to the results of Begg and Mazumdar’s test at a significance level of 0.1, no bias was observed in the results of the prevalence of osteoporosis in men worldwide in this study (*P* = 0.448). The analysis of the prevalence of osteoporosis by sex on each continent is reported in Table 3.

### Meta-regression test

**Fig. 6:**
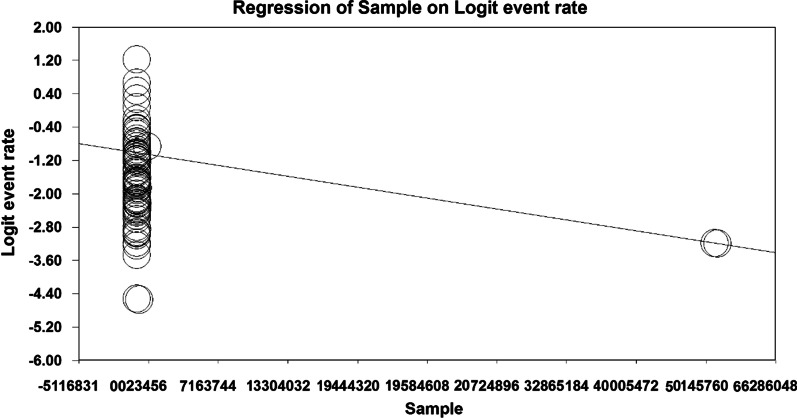
Meta-regression diagram of the prevalence of osteoporosis in the world by sample size

**Fig. 7:**
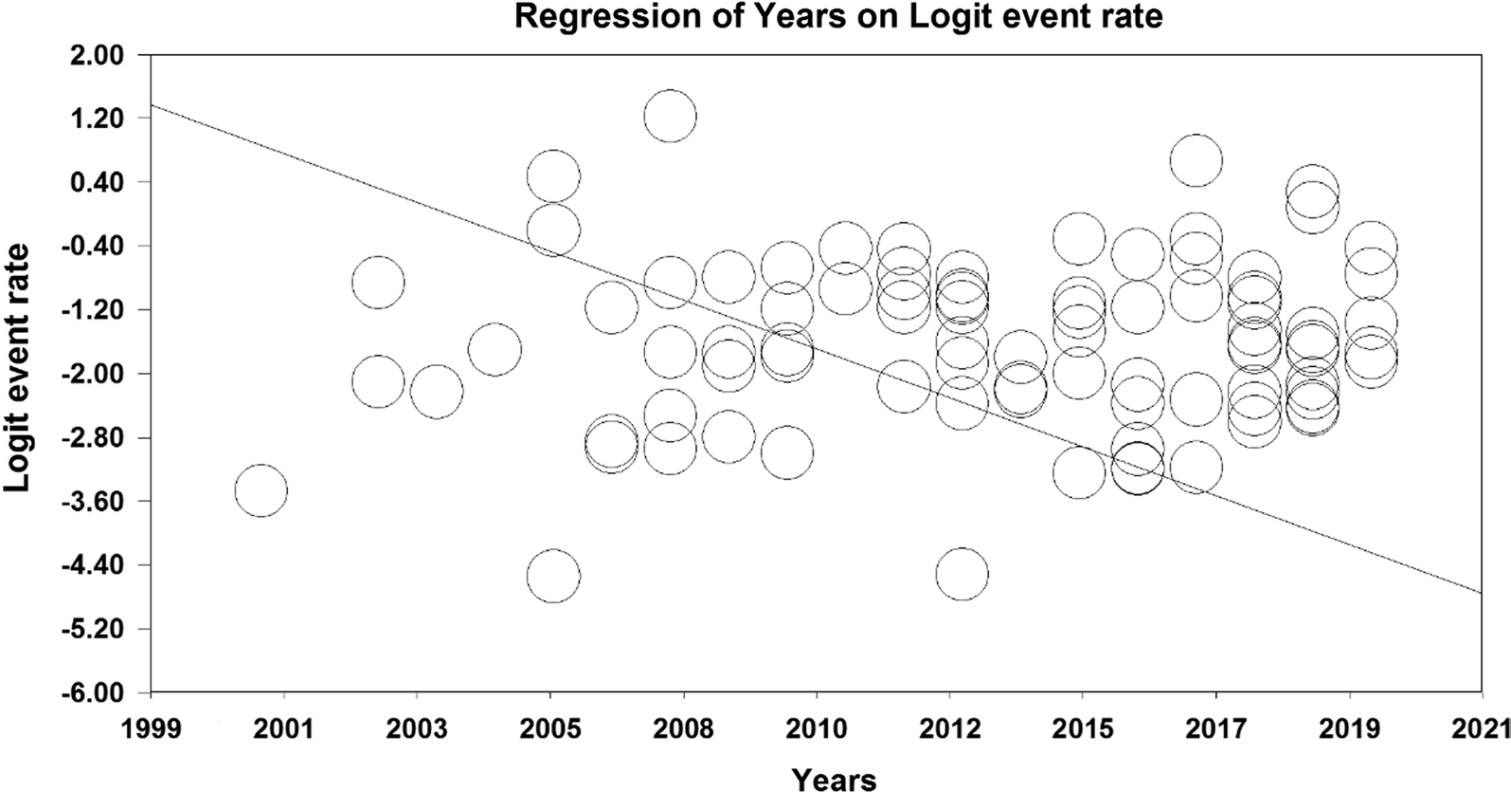
Meta-regression diagram of the prevalence of osteoporosis in the world by year of study

**Fig. 8:**
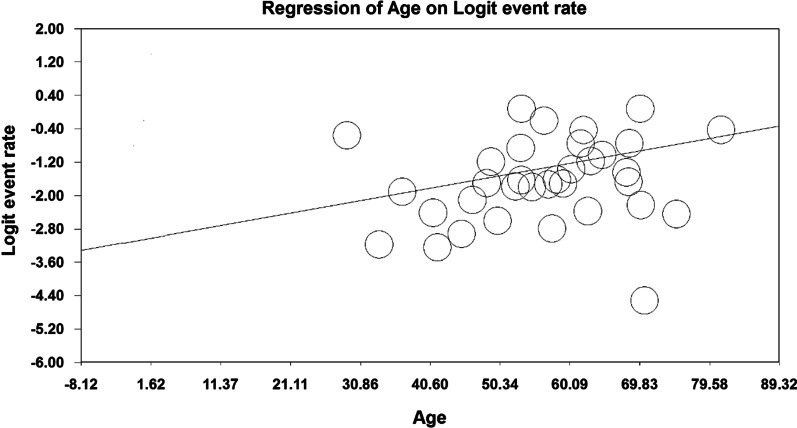
Meta-regression diagram of the prevalence of osteoporosis in the world by age of study participants

Given that the overall prevalence based on meta-analysis is influenced by factors such as sample size, year of research, age of study participants, place of study and gender, these factors increase heterogeneity and decrease the accuracy of results. Therefore, meta-regression analysis, as well as subgroup analysis, were used to examine the relationship between osteoporosis and this factors. Due to the effect of various factors in the incidence of heterogeneity between the results of osteoporosis studies globally, a meta-regression test was used to examine the effect of three factors: sample size, year of study, and age of the participants. According to Fig. 6, the prevalence of osteoporosis decreases with increasing the sample size, and this is statistically significant (*P* < 0.05). Moreover, Fig. 7 shows that the prevalence of osteoporosis decreases with increasing years of study, which is statistically significant (*P* < 0.05). The results reported in Fig. 8 show that the prevalence of osteoporosis studies in the world increases with age, which was also statistically significant (*P* < 0.05).

## Discussion

In this study, the prevalence of osteoporosis in the world was 18.3%, which is calculated based on reports of the prevalence of osteoporosis from 86 studies across five continents. Although the number of reported epidemiological studies on osteoporosis in Africa is limited, recent studies have shown that osteoporosis and related fractures increase across the continent [93]. Therefore, based on the results of this study, it was revealed that the prevalence of osteoporosis in Africa is much worse than that in other continents.

According to a systematic and meta-analysis study in China, the prevalence of osteoporosis from 2003 to October 2015 was reported to be 15.33% in men and 25.41% in women. It can be concluded that the overall prevalence of osteoporosis was 20% [94].

In a study, the prevalence of osteoporosis was assessed in several industrialized countries (USA, Canada, five European countries, Australia, and Japan) and people aged 50 and over. The prevalence of osteoporosis in the spine or hip was reported as follows: 26.3% in Japan, 21% in the USA, 14.3% in Germany, 9.9% in France, 9.7% in Italy, 7.8% in the United Kingdom, 6.3% in Spain, 2.6% in Canada, and 2% in Australia. Overall, the number of people with osteoporosis is estimated at 49 million [95].

In 2018, a systematic review and meta-analysis based on the World Health Organization (WHO) diagnostic criteria were conducted in the eastern Mediterranean: the study was conducted between 2000 and 2017 without any language restrictions; the prevalence of osteoporosis was 24.4%; the prevalence of osteoporosis is 24.4% in women and 20.5% in men [96].

The present study examined the PubMed, Science Direct, Web of Science, Scopus, Magiran, and Google Scholar databases that were searched with no lower time limit and until 2020. According to PRISMA checklist and flow chart, while Zamani et al. [96] studied only PubMed, Scopus, Web of Science, and Index Medicus for the EMR published between January 2000 and December 2017, we can say that the sensitivity of this study by examining more databases and finding more relevant studies is more than the study of Zamani et al. [96]. In addition, this study has been studied worldwide and by gender in all continents, but the study of Zamani et al. [96] has examined only the Eastern Mediterranean.

The prevalence of osteoporosis in women of the world was reported to be 23.1 (95% CI 19.8–26.9), and the prevalence of osteoporosis in men of the world was reported to be 11.7 (95% CI 9.6–14.1). The results of subgroup analysis also show that among men, the highest prevalence of osteoporosis was in Asia and among women, the highest prevalence of osteoporosis was in Africa, this is even though no studies have been conducted on men in the African continent and no African studies of men in the meta-analysis.

The highest prevalence of osteoporosis in the studies studied in Iran with 77.3% and the lowest prevalence in the Canadian study with 1.07% [30, 85]. Osteoporosis affects both males and females. Although the definition of osteoporosis is not necessarily associated with fractures, the unfortunate consequence is fractures [96–100]. The analysis showed that out of the diagnostic tools used to diagnose osteoporosis, the prevalence of osteoporosis was highest when diagnosed with BMD instruments.

According to a study in 1995 in the USA, approximately 1.5 million fractures are associated with osteoporosis each year. It is estimated that 80% of India’s urban population suffers from a deficiency of Vitamin D and hip fractures occur about a decade earlier than in Western nations. Therefore, osteoporosis is a major concern for this ageing population [101, 102].

Although there is no direct evidence that screening for osteoporosis reduces fractures, there is good indirect evidence that screening is effective in identifying post-menopausal women with low bone mineral density. Health policymakers can also help prevent and reduce osteoporosis in the community through a variety of means, such as moderate physical activity, an appropriate intake of calcium and vitamin D, cessation of smoking, and pharmaceutical intervention in high-risk groups. Also, effective dissemination of findings from research should be used to increase the awareness of osteoporosis, both among the general population and in the health services, to increase early detection of risk factors and to motivate preventive measures [90–102].

## Strengths and limitation

The most important strength of the present study is the comprehensive review of all databases, regular review of articles by three researchers and performing meta-regression and subgroup analysis to obtain more accurate information. The most important limitations of the present study were to encounter low-quality articles that had been published for years and their full text was not available for further review.

The present study aims to remove the limitations of systematic review studies and other meta-analyses in this field by using a comprehensive review of different sources, long time period, different meta-regression and subgroup analysis, and considering that articles in languages other than English and Persian were not considered and age-specific prevalence of osteoporosis were not reported, can be mentioned as limitations of this study.

## Conclusion

This study shows that the prevalence of osteoporosis in the world is very high, especially the prevalence in Africa and Europe is much higher and more significant. According to the medical, economic, and social burden of osteoporosis, providing a robust and comprehensive estimate of the prevalence of osteoporosis in the world can facilitate decisions in health system planning and policymaking, including an overview of the current and outlook for the future; provide the necessary facilities for the treatment of people with osteoporosis; reduce the severe risks that lead to death by preventing fractures.

## Data Availability

Datasets are available through the corresponding author upon reasonable request.

## Abbreviations

BMD: Bone mineral density
DXA: Dual-energy X-ray absorptiometry
SOS: Speed of sound
STROBE: Strengthening the Reporting of Observational Studies in Epidemiology for Cross-Sectional Study
PRISMA: Preferred Reporting Items for Systematic Reviews and Meta-Analysis
